# Acid-sensing ion channels in ischemic stroke: amplifying and constraining neuronal injury in a phase-specific framework

**DOI:** 10.1007/s00424-026-03217-5

**Published:** 2026-10-09

**Authors:** Octavia Yifang Weng, Jian-Fei Lu, Xin Qi, Xi Chen, Lu-Yang Wang, Tian-Le Xu

**Affiliations:** 1 Program in Neurosciences and Mental Health, The Hospital for Sick Children, 686 Bay Street, Toronto, ON M5G 0A4 Canada; 2 Program in Developmental, Stem Cell, and Cancer Biology, The Hospital for Sick Children, 686 Bay Street, Toronto, ON M5G 0A4 Canada; 3 Department of Physiology, University of Toronto, 1 King’s College Circle, Toronto, ON M5S 1A8 Canada; 4 Department of Anatomy and Physiology, Shanghai Key Laboratory of Emotions and Affective Disorders, Shanghai Jiao Tong University School of Medicine, Shanghai, 200025 China

**Keywords:** Acid-sensing ion channels, ASIC1a, Ischemic stroke, Acidotoxicity, Neuroprotection

## Abstract

For decades, ischemic neuronal death was understood largely as a story about glutamate. Acid-sensing ion channels (ASICs) have revised that account. When cerebral blood flow stops, the penumbra can develop prolonged extracellular acidosis, and experimental studies implicate ASIC1a in mediating sufficient Ca²⁺ entry to injure neurons even when glutamate receptors are blocked, a contribution shaped by channel expression, subunit composition, and the intracellular signaling context. However, sustained acidosis does not necessarily imply sustained channel opening, given ASIC1a desensitization. Besides acidosis, glutamate, polyamines, a paradoxical intracellular alkalization, and oxidative stress can enhance ASIC1a activity or expression, and the channel also signals through a non-conducting route, recruiting RIPK1 to trigger necroptosis independently of ion flux. The relative contributions of these conducting and non-conducting mechanisms in vivo remain incompletely defined. Endogenous mechanisms may constrain injury by internalizing the channel, altering subunit expression, including conditioning-associated ASIC2a upregulation, and degrading ASIC1a through estrogen signaling. We argue that these responses are better understood as constraints on injury than as a genuine survival program, a distinction with mechanistic and therapeutic consequences. Critically, the contribution of these mechanisms may vary over time. The same channel acts as a target of homeostatic regulation before stroke, a driver of death during ischemia, a potential target at reperfusion when acidosis persists but restored flow finally permits drug delivery, and an impediment to repair for weeks afterward. The question in ASIC-targeted neuroprotection is not only whether to inhibit the channel, but also when and for how long.

## Section I: ASICs in the context of stroke

### Stroke: why pH matters

Stroke ranks second among causes of death and third among causes of disability worldwide [26, 32]. Ischemic stroke, approximately 87% of cases, arises from cerebral arterial occlusion that deprives brain tissue of oxygen and metabolic substrate [54]. The narrow window for thrombolysis and thrombectomy [92] and the incomplete efficacy of neuroprotective strategies [13, 57] highlight the need to better understand ischemic neuronal death.

The physiological coupling between oxygen delivery and pH buffering collapses during ischemia. Stagnant red blood cells can neither offload CO₂ nor regenerate oxygenated hemoglobin, while ischemic cells shift to anaerobic glycolysis, producing lactic acid and H⁺ faster than residual buffering can absorb [68, 74]. Extracellular pH in the ischemic core falls to 6.0–6.5, and below 6.0 in complete ischemia with elevated glucose [37, 68]. Acidosis below pH 6.4 amplifies injury through denaturation of intracellular proteins, ASIC activation, and release of free ferrous iron [44, 50]. Surrounding the necrotic core is the penumbra, structurally intact but functionally compromised tissue that receives marginal perfusion [4, 38] and is maintained near pH 7.0 [35, 73]. Because penumbral neurons can recover if perfusion is restored, this tissue is the primary target for acute neuroprotection [35, 38].

Acidosis is a double-edged signal. Mild to moderate acidosis can be protective, reversibly suppressing neuronal excitability, delaying hypoxic spreading depolarization, and improving post-ischemic recovery [55, 71], while inhibiting Ca²⁺-dependent vesicular glutamate release [25] and attenuating excitotoxic injury [72]. When pH drops lower and is sustained, acidosis transitions from an adaptive brake to a lethal accelerator. Among the sensors of this pathological acidosis, ASICs are well positioned, not as passive transducers but as integrators that steer the balance toward death or survival.

### ASICs: subunits and functional diversity

ASICs are voltage-independent, proton-gated cation channels belonging to the degenerin/epithelial sodium channel (DEG/ENaC) superfamily. In mammals, four genes encode at least six subunit proteins, including ASIC1a [77], ASIC1b [15], ASIC2a [76], ASIC2b [49], ASIC3 [78], and ASIC4 [2], which assemble as homotrimers or heterotrimers with distinct biophysical properties [40, 63, 67]. CNS expression is dominated by ASIC1a and ASIC2a, with regional and cell-type differences.

Subunit composition determines both the pH sensitivity and ion permeability of ASIC channels. ASIC1a homotrimers are activated near pH 7.0, though the precise value depends on conditioning pH and recording conditions, and are permeable to Ca²⁺ in addition to Na⁺ and K⁺ [7, 77, 85]. Homomeric ASIC1b channels, formed from an alternatively spliced product of the ASIC1 gene predominantly expressed in peripheral sensory neurons, respond with similar kinetics but are impermeable to Ca²⁺ in rodents [7, 15]. ASIC2a, expressed in both peripheral sensory and central neurons, is less proton sensitive, with homomers half-maximally activated near pH 4.5 [36], well below penumbral pH. This does not exclude a role for ASIC2a heteromers during ischemia, since pairing with ASIC1a shifts proton sensitivity into the pathophysiological range. ASIC2b is non-functional as a homomer but modulates channel activity within heteromers [36, 66]. ASIC1a homomers are therefore positioned to transduce penumbral acidosis into intracellular Ca²⁺ signals, whereas ASIC2a-containing heteromers, with reduced proton sensitivity [36] and low Ca²⁺ permeability, may mediate Ca²⁺-dependent acidotoxicity less effectively [28, 67]. Whether ischemia-associated changes in subunit expression produce such a functional shift in native neurons remains unresolved. Zn²⁺, an endogenous modulator, acts bidirectionally and subunit-specifically. Micromolar Zn²⁺ potentiates acid activation of ASIC2a-containing channels [5], whereas nanomolar Zn²⁺ inhibits ASIC currents, an effect confined to ASIC1a homomers and ASIC1a-2a heteromers [18]. Acid-evoked Zn²⁺ currents have also been reported in recombinant ASIC2a homomers and ASIC1a/2a heteromers, but not ASIC1a homomers, at pH 5.0 [19]. In cultured cortical neurons, Zn²⁺ enhanced injury under severe acidification (pH 4.0), indicating that low Ca²⁺ permeability does not necessarily imply low acidotoxic potential. The contribution of this pathway to ischemic injury in vivo remains to be established. These properties reflect subunit-specific differences in extracellular architecture and gating. Six major conformations of human ASIC1a captured by cryo-EM from pH 8.5 to 5.7 provide a structural blueprint for its dynamic response to acidification [10].

In addition to their role as proton-gated ion channels, ASICs, and ASIC1a in particular, are increasingly recognized as scaffolds that engage intracellular signaling independent of ion conduction.

### ASIC activation links sustained ischemic acidosis to neuronal injury

Physiologically, ASIC1a is activated transiently by pH transients from synaptic vesicle release or mild local acidosis, then desensitizes rapidly [20, 33]. The ischemic microenvironment instead sustains acidosis: anaerobic metabolism, impaired CO₂ clearance, and failing pH-buffering mechanisms maintain penumbral acidosis for minutes to hours [68, 69]. Whether sustained mild acidosis translates into sustained ASIC1a signaling is less clear, since progressive acidification promotes steady-state desensitization rather than opening. Nonetheless, ASIC1a activity under these conditions correlates with accumulating Ca²⁺ influx and markers of neuronal injury [58, 85]. Several mechanisms could account for this. Brief opening may initiate injury that continues after desensitization. Residual currents and repeated activation during pH fluctuations may also contribute, although the latter requires sufficient recovery from desensitization. Alternatively, ischemic co-signals, including glutamate release, polyamine efflux, and intracellular alkalization may amplify ASIC1a activity beyond what acidosis alone produces. Zinc is a further ischemic co-signal with an unclear net effect. It enters neurons through NMDA receptor-gated channels [41], and its contribution depends on subunit composition and Zn²⁺ concentration, as discussed in Section “ASICs: subunits and functional diversity”. Surface ASIC1a may also increase following serum or insulin withdrawal in experimental preparations [12], although increased surface availability alone does not establish sustained opening under acidic conditions, and signaling that does not require ion flux may also contribute [80]. These mechanisms are supported by different levels of evidence and might predict different relationships between current and injury.

The NMDA receptor field offers an instructive precedent. NMDA receptor activation can be physiologically essential, supporting synaptic plasticity, learning, and memory, or profoundly excitotoxic, depending on subunit composition, subcellular localization, and the duration and intensity of activation [34, 51]. NR2A-containing receptors drive pro-survival signaling while NR2B-containing receptors drive cell death programs [16, 51]. Independently, synaptic NMDARs promote survival while extrasynaptic NMDARs promote death [34]. ASICs occupy an analogous position. The same channel family, with the same activating stimulus, can mediate opposing outcomes. The variables that may shape ASIC1a-dependent outcomes are discussed in Section “Variables that shape ASIC1a outcomes, and what remains unresolved”.

## Section II: ASIC1a-mediated injury and its endogenous constraint

**Fig. 1:**
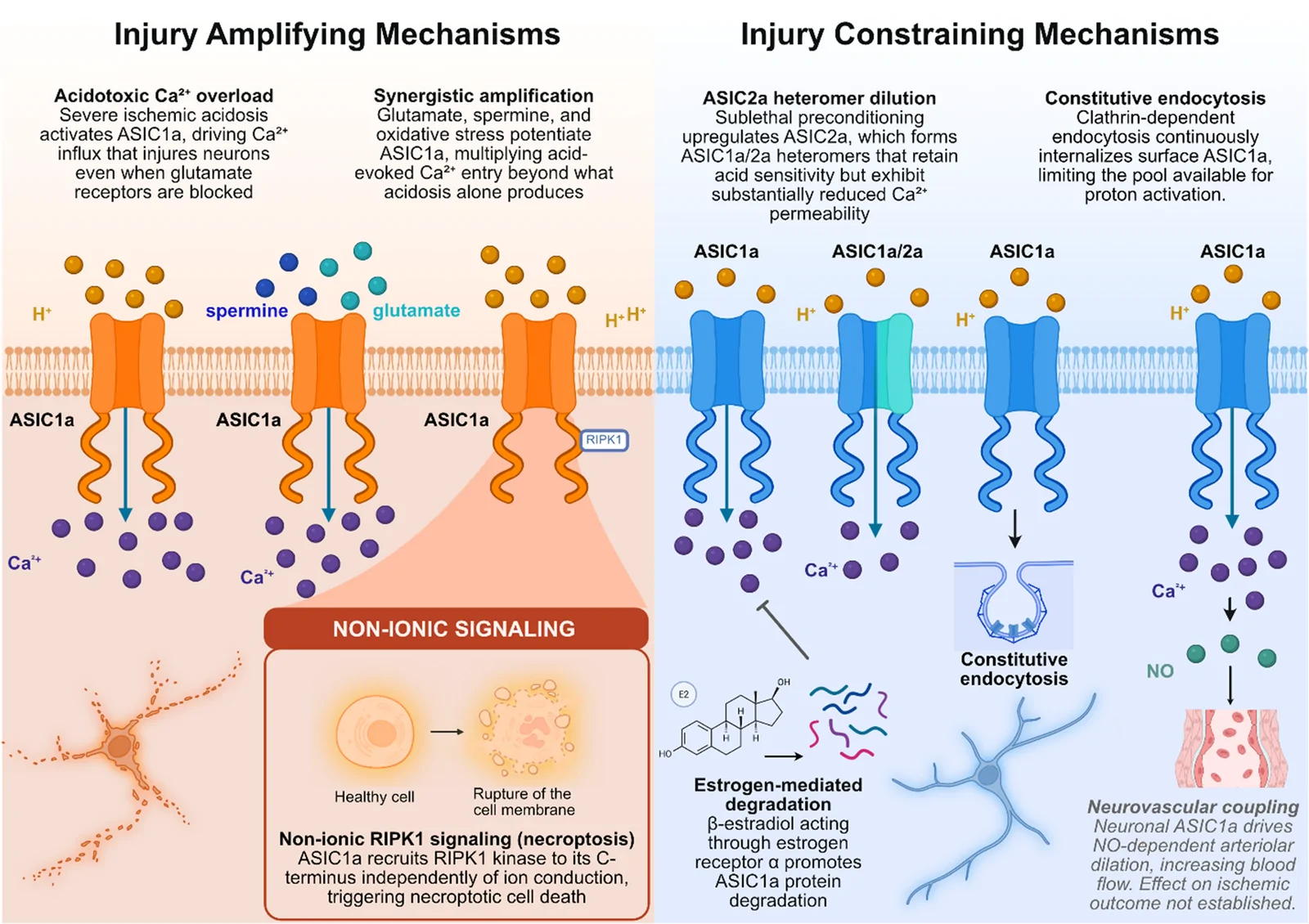
ASIC1a-mediated injury and the endogenous mechanisms that constrain the injury. Injury-amplifying mechanisms (left) include acidotoxic Ca²⁺ overload through ASIC1a, potentiation of channel activity by glutamate, spermine, and oxidative stress, and a non-ionic signaling arm in which ASIC1a recruits RIPK1 to its C-terminus to initiate necroptosis independently of ion conduction. Injury-constraining mechanisms (right) include constitutive clathrin-dependent endocytosis that limits surface ASIC1a, ischemic preconditioning–induced ASIC2a upregulation that may form heteromers with reduced Ca²⁺ permeability, and β-estradiol–driven, ERα-dependent degradation of ASIC1a protein. Set apart at right, and distinct from these constraining mechanisms, is ASIC1a-dependent neurovascular coupling, in which neuronal ASIC1a drives nitric oxide-dependent dilation of cerebral arterioles. This represents active signaling by the channel rather than limitation of its availability, and its effect on ischemic outcome has not been established. Throughout both figures, elements rendered in italic grey font rest on mechanistic interpretation, extrapolation across preparation or species, or proposed therapeutic opportunity, whereas elements in black are supported by direct experimental evidence in a relevant model. This image is created with biorender and affinity designer: https://BioRender.com/gadhzhv

The relationship between ASIC activation and ischemic neuronal death is not linear. As discussed in Section “Section I: ASICs in the context of stroke”, injury arises through co-signals that amplify ASIC activity, altered ASIC surface expression, and downstream death pathways. Endogenous mechanisms also constrain surface expression, modify subunit composition and may thereby limit ASIC1a-mediated injury. The competing roles are summarized in Fig. 1.

### ASIC1a-mediated neuronal injury

#### Acidotoxicity: Ca²⁺ overload independent of glutamate

The canonical model of ischemic neuronal death centers on NMDAR-mediated glutamate excitotoxicity. This view was challenged by evidence that ischemic death can occur through a distinct, NMDAR-independent pathway driven by ASIC1a-mediated Ca²⁺ overload. Glutamate, and even NMDAR antagonists such as AP5, were later shown to potentiate ASIC1a currents directly, suggesting that glutamate-targeted neuroprotection with NMDAR blockade alone may not be sufficient [43, 85]. Lowering extracellular pH to ischemic levels activates amiloride-sensitive ASIC currents in most cultured mouse cortical neurons and drives Ca²⁺-dependent injury even with voltage-gated Ca²⁺ channel and glutamate receptor blockers present [85]. Pharmacological blockade of ASIC1a or deletion of *Asic1a* conferred neuroprotection in mouse focal ischemia, more potently than glutamate antagonism alone, establishing acidotoxicity as a parallel rather than a secondary injury mechanism [58, 85].

#### Synergistic amplification by ischemic co-signals

Multiple ischemic signals converge on ASIC1a, potentiating activity well beyond what acidosis alone produces. Glutamate released from depolarizing neurons binds a distinct extracellular cavity on ASIC1a as a positive allosteric modulator, increasing proton affinity and open probability and aggravating neurotoxicity in vitro and in vivo [43]. Glutamate also acts indirectly. NR2B-containing NMDAR activation drives CaMKII-mediated phosphorylation of ASIC1a at Ser478 and Ser479, sensitizing it to acidosis, as shown in rat global ischemia models and hippocampal cultures [30]. These interactions indicate that glutamatergic and ASIC1a-dependent injury mechanisms are not entirely independent [39]. Extracellular spermine, released from dying cells, sensitizes ASIC1a to acidosis and exacerbates ischemic injury in mouse focal ischemia [21]. Paradoxical intracellular alkalization in the penumbra, rather than the assumed acidification, potentiates ASIC1a gating and worsens injury in cultured neurons and mouse focal ischemia [45]. Additionally, oxidative stress upregulates ASIC1a expression through a JNK-dependent mechanism in a neuronal cell line and cortical cultures, linking ROS generation to increased channel activity [84]. Together these findings suggest ASIC1a’s contribution reflects modulation by multiple ischemia-associated factors, although their combined effects during ischemia remain to be established.

#### Non-ionic death signaling: beyond ion conduction

ASIC1a can also mediate neuronal death without ion permeation through the pore. Tissue acidosis induces necroptosis via ASIC1a acting as a molecular signaling scaffold, recruiting receptor-interacting protein kinase 1 (RIPK1) to the C-terminus and triggering RIPK1 phosphorylation and neuronal death independently of ionic conductance, as demonstrated in cultured mouse cortical neurons [80, 81]. In a mouse model of focal ischemia, middle cerebral artery occlusion (MCAO) produced ASIC1a-RIPK1 association and RIPK1 phosphorylation in the affected hemisphere, both largely prevented by *Asic1a* deletion [80]. Therapeutically, inhibition of ionic current should not be assumed to predict inhibition of conformational signaling. PcTx1, which acts at the extracellular domain, also inhibited acid-induced ASIC1a-RIPK1 association and RIPK1 phosphorylation in cultured neurons [80]. Whether combining channel inhibition with disruption of the ASIC1a-RIPK1 axis produces additive or synergistic neuroprotection remains open.

#### ASIC activity in non-neuronal cells: injury amplification beyond neurons

ASICs are expressed and functional in non-neuronal cells, suggesting contributions to stroke pathology beyond neuronal injury. Astrocytic ASIC1a is implicated in chronic epileptogenesis following injury, as shown in pilocarpine-induced status epilepticus in mice and in resected human epileptic hippocampal tissue [46, 88]. Cultured neonatal rat oligodendrocytes express ASIC1a, ASIC2a, and ASIC4 transcripts, each several-fold more abundant in OPCs than in mature oligodendroglia, with acid-evoked Ca²⁺ responses largely confined to the progenitor stage [27], offering a plausible route to white-matter injury and myelin loss. Microglial ASIC1 and ASIC2a are upregulated by inflammatory stimulation, and their blockade reduces cytokine production and microglial migration, although this was shown in cultured rat microglia activated with lipopolysaccharide rather than by ischemia [28, 89]. Ischemic acidosis may therefore extend beyond neuronal acidotoxicity to glial and immune-mediated amplification, though the evidence in each case comes from non-ischemic models, as examined further in Section “Non-neuronal cell contributions to ASIC-mediated stroke pathology”.

### Endogenous mechanisms that constrain ASIC1a-mediated injury

Before reviewing this literature, it is important to distinguish two conceptually distinct processes: injury constraint (reducing channel availability or activity) versus active pro-survival signaling (in which ASIC isoforms directly trigger protective pathways). Most mechanisms reported in the CNS, including endocytosis and degradation, fall into the first category. Genuine ASIC-driven survival signaling, analogous to the PI3K-AKT pathway engaged by synaptic NMDARs, remains underexplored.

Neurons limit the injurious potential of surface ASIC1a through constitutive clathrin- and dynamin-dependent endocytosis, which reduces the membrane pool and thereby limits acid-evoked Ca²⁺ influx. Experimental blockade of this endocytic process exacerbates acidosis-induced neuronal death in cultured rat and mouse cortical neurons, demonstrating that constitutive removal is a meaningful brake on acidotoxicity [90]. A distinct, phosphorylation-dependent route can also augment surface ASIC1a, whereby BDNF acting through TrkB engages a PI3K/Akt cascade that phosphorylates ASIC1a at Ser25 and drives its forward trafficking to the membrane of spinal dorsal horn neurons [22].

A second route is subunit remodeling. Ischemic preconditioning, in which a brief sublethal ischemic episode confers protection against a subsequent lethal insult, remodels the ASIC landscape toward a more protective configuration. In a four-vessel occlusion model, a three-minute sublethal episode 48 h before a lethal 15-minute insult protected hippocampal CA1 neurons, and was associated with upregulation of ASIC2a mRNA and protein in CA1 neurons relative to lethal ischemia alone [56]. Because ASIC2a-containing heteromers are less Ca²⁺ permeable than ASIC1a homomers in recombinant systems [36, 67], this shift could reduce vulnerability to acidotoxicity. In rat focal ischemia, preconditioning and postconditioning both reduce ASIC1a expression in the temporoparietal cortex through a p-AKT-dependent pathway [59]. Whether ASIC2a upregulation and ASIC1a downregulation are independent or coordinated remains unresolved.

The proposed protective effect of ASIC2a incorporation is based largely on the biophysical properties of recombinant channels. Preconditioning raises ASIC2a mRNA and protein, but has not been shown to produce heteromers or to change acid-evoked currents in CA1 neurons [56]. ASIC2a is better described as a subunit that changes how ASIC1a behaves, and PcTx1 illustrates this well, inhibiting homomeric ASIC1a while potentiating ASIC1a/2a heteromers in cortical neurons cultured from rat and mouse under the same conditions [52]. The exact conditions under which ASIC2a upregulation renders neurons less vulnerable to ischemic acidosis remain to be defined.

Hormonal signaling provides a further route. Beta-estradiol protects against acidosis-mediated and ischemic neuronal injury by promoting ASIC1a protein degradation in an estrogen receptor α-dependent manner, linking sex hormone signaling to channel availability, as shown in cultured mouse cortical neurons and mouse focal ischemia [91]. This estrogenic control is not uniform across cell types, however, as β-estradiol conversely rapidly potentiates ASIC currents in peripheral sensory neurons [62]. If the effect holds at physiological estradiol levels, treatments could potentially enhance ASIC1a degradation rather than block the channel directly.

#### Active ASIC pro-survival protection: a distinct and largely untested possibility

Evidence for ASIC isoforms actively driving pro-survival signaling remains largely indirect, and the most suggestive findings lie outside the canonical neuronal acidotoxicity pathway. One candidate is the coupling of ASIC activity to cerebral blood flow. Neuronal ASIC1a is required for CO_2_-evoked, nitric oxide–dependent dilation of cerebral arterioles in vivo, including in mice with neuron-specific ASIC1a knockouts, positioning the channel as a sensor matching perfusion to local acidosis [24]. Vascular ASICs may also contribute, though the direction is less clear. ASIC-1, -2, and − 4 expression has been documented in the rat middle cerebral artery, yet ASIC blockade augmented rather than suppressed acidosis-induced relaxation of isolated arterial segments [1]. Whether these vasomotor actions are net protective during ischemia is unresolved.

A second candidate is ASIC3. Unlike other family members, ASIC3 generates a sustained current within the narrow pH range of cardiac and skeletal-muscle ischemia, suited to peripheral rather than cerebral acidosis, as characterized in rat cardiac sensory neurons and recombinant channels [70, 87]. Loss of ASIC3 in mice worsens cardiac injury after experimental ischemia induced by isoproterenol, consistent with a protective sensor role [17]. Whether these peripheral findings extend to cerebral ischemia remains an open question.

ASIC-driven pro-survival protection in ischemic stroke is best regarded as a hypothesis rather than an established mechanism. Plausible mechanisms exist, but their protective contributions have not been established at specific stages of ischemic injury, in particular the hyperacute window, when acidosis, perfusion, and channel surface expression change most rapidly. Defining when, rather than only whether, ASIC activity becomes protective is the central open question.

### Variables that shape ASIC1a outcomes, and what remains unresolved

ASICs can drive acidotoxicity while endogenous regulation can constrain their contribution to injury. As with NMDARs (see Section “ASIC activation links sustained ischemic acidosis to neuronal injury”), the outcome depends on how the channel is engaged, not whether it is present. Four variables are relevant. The first is the degree and duration of acidosis. Mild acidification protects hippocampal neurons against oxygen-glucose deprivation in culture [72], delays hypoxic spreading depression in adult rat hippocampal slices [71], and suppresses presynaptic glutamate release in rat brain synaptosomes [25]. These protective effects of acidosis do not themselves establish a protective role for ASIC activation. Acidosis sustained for hours in the penumbra does the opposite [68, 69]. The second is subunit composition. Ischemic preconditioning upregulates ASIC2a [56], shifting the channel population toward lower proton sensitivity [36] and reduced Ca²⁺ permeability [28, 67]. The third is how much ASIC1a reaches the surface, set by constitutive endocytosis [90], ischemia-driven trafficking to the membrane [12], and Akt-dependent downregulation [59]. The fourth is phosphorylation, which can influence both gating and trafficking. CaMKII sensitizes the channel to acidosis [30], while BDNF-TrkB signaling drives more channel to the membrane [22].

Less is established than this list suggests. Ischemic conditioning changes ASIC2a and ASIC1a expression [56, 59], but whether these two changes are coordinated is unknown. The role of Akt is also unclear, since sustained Akt activation lowers ASIC1a and protects [59] while BDNF-driven Akt signaling raises surface ASIC1a levels [22]. Most importantly, the cited conditioning studies do not establish whether ASIC1a activation is required to trigger tolerance. Constitutive *Asic1a* deletion alone cannot distinguish a role in initiating tolerance from reduced susceptibility to the subsequent lethal insult. Temporally restricted inhibition during conditioning, with drug washout before the lethal insult, could help separate these possibilities. For now, ASIC1a is better described as a target of endogenous tolerance than as a trigger.

## Section III: temporal dynamics of ASIC-mediated injury and its constraint across the stroke continuum

Section “Section II: ASIC1a-mediated injury and its endogenous constraint” establishes that ASICs participate in a counter-balance between injury amplification and endogenous mechanisms that constrain it. This balance is not fixed, which shifts as the cellular and extracellular environment changes from the pre-stroke state through acute ischemia, reperfusion, and into the weeks of post-stroke recovery.

**Fig. 2:**
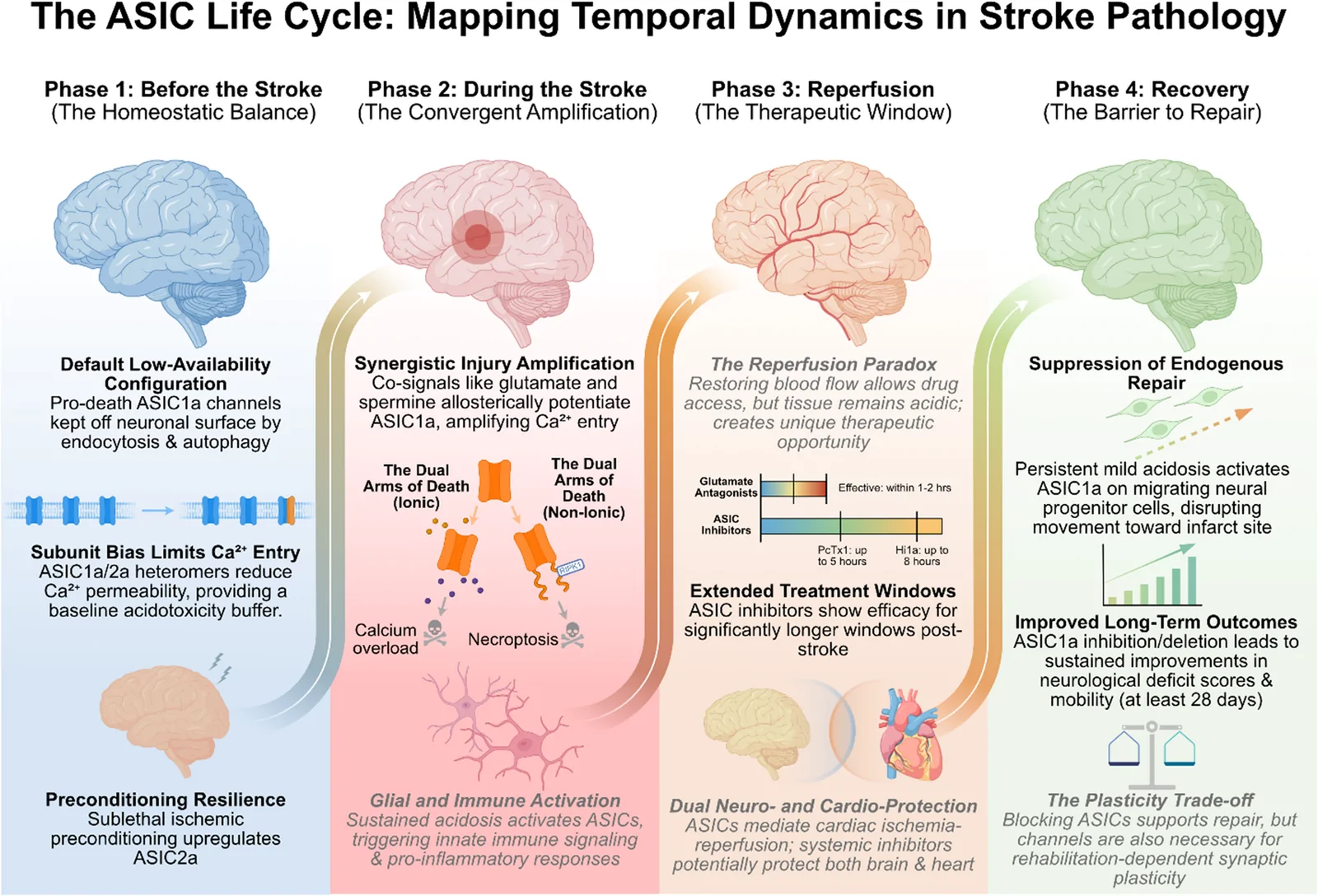
The ASIC life cycle across stroke pathophysiology. Phase 1 (pre-stroke): ASIC1a surface expression is limited by constitutive endocytosis, ASIC2a-containing heteromers are expected to reduce Ca²⁺ entry, and preconditioning further lowers injury potential through ASIC2a upregulation and Akt-dependent ASIC1a downregulation. Phase 2 (acute ischemia): glutamate, spermine, and oxidative stress amplify ASIC1a activity, driving parallel ionic (Ca²⁺ overload) and non-ionic (RIPK1-dependent necroptosis) death pathways; glial and immune activation is shown as a proposed additional contribution, since the supporting evidence derives from non-ischemic models. Phase 3 (reperfusion): ASIC inhibitors retain efficacy at substantially longer post-occlusion intervals than glutamate antagonists in preclinical models, though the agents shown were administered intracerebroventricularly; the attribution of these extended windows to persistent penumbral acidosis, and the possibility of combined neuroprotection and cardioprotection from a single systemic agent, are shown as interpretations rather than established findings. Phase 4 (recovery): ASIC1a activation on migrating neural progenitors impairs endogenous repair, and ASIC1a inhibition or deletion improves neurological deficit scores and mobility over 28 days. The requirement to balance sustained inhibition against ASIC-dependent synaptic plasticity is shown as a proposed trade-off. As in Fig. 1, black indicates direct experimental support in a relevant model, while grey italics indicate inference, extrapolation, or proposal. This image is created with biorender and affinity designer: https://BioRender.com/yrpgocd

The framework below derives entirely from preclinical work. Clinical efficacy of ASIC-targeted treatment in stroke patients remains unestablished. The human evidence discussed here consists of expression and functional studies in resected or post-mortem tissue and to the species comparisons in Section “Human versus rodent ASIC: translational considerations”. Stages here are defined by the dominant pathophysiological process at each point rather than by conventional clinical staging [8]. The cited recovery framework groups the first 24 h into a single hyperacute phase, so the events determining the contribution of ASICs to ischemic injury are compressed within a single clinical interval. Reperfusion, which may improve drug delivery and alter the context of ASIC-targeted intervention, is not itself a clinical phase boundary. A pathophysiologically defined timeline is therefore needed to resolve when, and by what mechanism, ASIC-mediated injury is constrained or unleashed. The phases below organize preclinical findings mechanistically rather than prescribe a treatment schedule, and how they map onto clinical intervals remains to be established. The four phases are summarized in Fig. 2.

### Before stroke: mechanisms that limit ASIC1a-dependent injury

In the healthy brain, homeostatic mechanisms converge to constrain injurious ASIC signaling, establishing a default configuration in which acidotoxic capacity is minimized. Constitutive clathrin- and dynamin-dependent endocytosis removes ASIC1a from the neuronal surface [90], estrogen receptor α-dependent degradation further limits the surface pool [91], and the default CNS subunit composition, abundant in ASIC2a-containing heteromers, may reduce Ca²⁺ entry [28, 67]. ASIC1a may contribute an active protective function in the intact brain, since neuronal ASIC1a is required for CO₂- and acidosis-evoked, nitric oxide–dependent cerebral vasodilation that couples local perfusion to metabolic demand [24].

This pre-stroke regulation of ASIC1a availability is not static. Ischemic preconditioning remodels it durably, upregulating ASIC2a expression in vulnerable hippocampal neurons [56] and downregulating ASIC1a through an Akt-dependent pathway [59], as discussed in Section “Variables that shape ASIC1a outcomes, and what remains unresolved”. Promoting ASIC2a expression, enhancing ASIC1a endocytosis, or leveraging hormonal degradation would all act on channels that participate in normal synaptic plasticity, learning, sensory processing, and cerebral vascular regulation [24, 83], and none of these approaches has been tested for safety or specificity. The central question is whether ASIC1a or ASIC2a can be modulated selectively and transiently enough to produce a protective state without disrupting these functions. Until that is addressed, the preconditioning findings provide a rationale for further experimental work rather than a basis for preventive treatment in any patient group.

### During ischemia: endogenous constraints are overwhelmed

Ischemia does not simply activate ASIC channels in isolation. It generates convergent co-signals that synergistically amplify ASIC1a activity far beyond any single input. Glutamate released from depolarizing neurons potentiates ASIC1a through direct allosteric binding [43], extracellular spermine sensitizes this channel to acidosis [21], intracellular alkalization further modulates channel gating [45], and oxidative stress drives ASIC1a protein upregulation via the JNK pathway [84]. These emerge within minutes of occlusion, so ASIC1a activity during real ischemia reflects the multiplicative product of all inputs rather than their sum. The pre-stroke brakes, constitutive endocytosis, hormonal degradation, and potential modulation by ASIC2a-containing heteromers, may be insufficient to prevent injury under these conditions.

The non-ionic arm, in which acid-induced conformational change recruits RIPK1 to initiate necroptosis independently of ion conduction [80, 81], may also contribute to injury during this phase. Non-neuronal amplification may further extend the cascade through ASIC-dependent glial responses, although the supporting evidence derives mainly from non-ischemic models and the contribution of these responses during stroke remains to be established [28, 89].

The acute phase is where ASIC blockade has the most firmly established preclinical benefit. Intracerebroventricular PcTx1 reduced infarct volume by more than 50% when given up to five hours after MCAO in mice, with protection persisting at least seven days after transient MCAO. Protection was also demonstrated in permanent occlusion models [58]. Translation is limited mainly by delivery to ischemic tissue when perfusion is compromised, though blood-brain barrier-permeable small molecules are progressing: C5b, a PcTx1-inspired orthosteric ASIC1a antagonist from structure-based design [9], retains efficacy following intravenous administration in MCAO models [60].

The gap to clinical use remains wide. PcTx1 and Hi1a were both given intracerebroventricularly [14, 58], a route unavailable in stroke care, and their extended windows were established under that condition. Intranasal PcTx1 has also shown neuroprotective efficacy [3, 60]. C5b has demonstrated efficacy after intravenous pretreatment, with reported plasma pharmacokinetics and brain exposure, including an elimination half-life of approximately 6.5 h and detection in brain tissue within 2 min of dosing [60]. Its efficacy when administered after stroke onset remains to be established. Pharmacological profiles also differ. PcTx1 potentiates ASIC1a/2a heteromers while inhibiting ASIC1a homomers, and Hi1a blocks ASIC1a incompletely. Prolonged inhibition may also carry costs, as ASIC1a contributes to hippocampal LTP [83] and to cerebral vasodilation [24]. ASIC1a inhibition is therefore a promising preclinical strategy whose translation depends on further characterization of pharmacokinetics, target engagement, safety, and efficacy in clinically relevant stroke models, including assessment across age, sex, and common comorbidities.

### Reperfusion: a pivotal window for pharmacological intervention

During occlusion, drug delivery to penumbral tissue is limited by compromised perfusion. At reperfusion, blood flow and drug access are restored, but tissue remains acidic because anaerobic metabolites are not immediately cleared and metabolic dysfunction persists.

This coincidence may account for the extended therapeutic windows of ASIC1a inhibitors relative to glutamate antagonists. Glutamate surges occur within minutes of reperfusion, whereas penumbral acidosis is maintained for hours by ongoing anaerobic metabolism and impaired buffering [74]. PcTx1 retains neuroprotective efficacy at up to five hours post-occlusion [58], while Hi1a, a more potent inhibitor that stabilizes ASIC1a in the closed state by a pH-independent mechanism, conferred neuroprotection as late as eight hours after stroke induction in rats [14]. By contrast, glutamate antagonists are generally effective only within one to two hours of insult onset [42]. This extended window is consistent with persistent penumbral acidosis, but the hypothesis has not been tested directly, and tissue pH, reperfusion, and metabolic recovery evolve together. The valence of acidosis at reperfusion depends on duration. Minutes of moderate acidification can be protective: brief CO₂ inhalation shortly after reperfusion reduces infarct volume in mice, and buffering brain pH with NaHCO₃ abolishes the protection conferred by ischemic postconditioning [23]. The hours of sustained penumbral acidosis described above may act through a different regime, so the more appropriate target at this phase is ASIC1a itself rather than the acidosis that activates it.

Combinatorial strategies are particularly attractive at this phase. ASIC1a and NMDAR blockade target distinct but interacting injury pathways, and combining PcTx1 with NMDAR antagonism produces additive neuroprotection, with PcTx1 extending the effective window of NMDAR-directed therapy [58]. Whether this translates to clinically relevant conditions remains untested. The available evidence is preclinical, and synergy in patients requires further investigation.

The reperfusion phase is relevant to the heart as well as the brain. ASIC1a-mediated injury occurs in cardiac ischemia-reperfusion models, and Hi1a or PcTx1 given at reperfusion onset conferred substantial cardioprotection [64]. Given the prevalence of cardiac comorbidity in stroke patients, a systemically delivered ASIC1a inhibitor at reperfusion could in principle provide simultaneous neuro- and cardioprotection, an opportunity few candidates offer, though this has not been tested in animals carrying the comorbidities typical of stroke patients.

### Recovery: ASICs continue to shape outcomes for weeks

ASICs continue to shape neural progenitor cell biology and functional recovery over weeks to months, and may also contribute to chronic neuroinflammation. The first possibility involves ASIC1a signaling in progenitors migrating from the subventricular zone toward the infarct. Following ischemia, these cells proliferate and migrate toward the penumbra as part of an endogenous repair response. ASIC1a is expressed in these migrating cells, and experiments in cultured progenitors link acid-induced ASIC1a activation to RhoA signaling that disrupts filopodia formation and cytoskeletal reorganization and impairs both migration and subsequent neurogenesis [31]. Pharmacological inhibition or genetic deletion of ASIC1a restored neural progenitor migration and improved functional recovery in the weeks following experimental stroke [31]. In this model, ASIC1a may impair recovery not only through its acute injury-promoting role but through a distinct, delayed mechanism that limits the brain’s endogenous repair capacity.

If ASIC1a limits repair in this way, its inhibition should improve outcomes over weeks rather than days. Both ASIC1a knockout and a single intranasal dose of PcTx1 administered 30 min after MCAO improved neurological deficit scores and open-field mobility over 28 days after experimental stroke, demonstrating that early inhibition can yield lasting functional benefits [3]. These results do not distinguish benefits arising from reduced acute injury from direct effects on later repair. Because recovery depends heavily on synaptic plasticity and circuit remodeling in peri-infarct cortex, the role of ASIC1a in NMDA receptor-dependent long-term potentiation [82] raises the unresolved question of whether sustained inhibition, while acutely protective, might dampen the plasticity that drives behavioral recovery.

A second, less established possibility is chronic neuroinflammation. Ischemic neuronal death releases damage-associated molecular patterns that activate innate immunity, and microglial ASICs are functional and upregulated by inflammatory stimulation [28, 89]. Whether the pH in the peri-infarct zone remains low enough, and long enough, to sustain ASIC activation on these cells has not been shown, and no causal link between ASIC activation and a persistent pro-inflammatory phenotype has been established. Testing such a mechanism would require direct peri-infarct pH measurement alongside cell-type-specific manipulation of glial ASICs.

### Evidentiary status of the framework

Across four phases, the evidence is uneven. The three most supported claims, which have been reproduced across laboratories, models, and species, are that acidosis reaching the depth and duration observed in the penumbra activates ASIC1a; that ASIC1a-containing channels are Ca²⁺-permeable in a subunit-dependent manner; and that genetic deletion or acute blockade of ASIC1a reduces infarct volume in rodent focal ischemia. Much of what follows is conditional on experimental context.

The pharmacological evidence deserves scrutiny. PcTx1 is treated as a selective ASIC1a antagonist, but it inhibits homomers only within a narrow range of conditioning pH and potentiates ASIC1a/2a heteromers at physiological pH by increasing their apparent proton affinity [52]. Because ASIC1a/2a heteromers are abundant in cortical neurons, the net action of PcTx1 on native brain ASICs is not straightforwardly inhibitory, and neuroprotection obtained with it should not be read as a clean measure of ASIC1a pore blockade. That said, three pharmacologically distinct compounds, PcTx1, Hi1a and C5b, all reduce neuronal injury, providing convergent pharmacological support for ASIC1a as a relevant target, particularly when considered alongside genetic evidence.

Meanwhile, *Asic1a* deletion removes the channel from every compartment, including the mitochondrial pool that regulates permeability transition, which extracellularly applied inhibitors may not reach [79]. Deletion and blockade are therefore not interchangeable manipulations, and where their results agree they may agree for different reasons. Deletion is also not a complete elimination of acid sensing, since ASIC1a-deficient neurons retain ASIC2a-containing channels that activate at stronger acidification [63]. Constitutive knockouts further permit lifelong compensation, making it difficult to separate developmental adaptations from the acute contribution of ASIC1a.

The endogenous-constraint arm is the most cell-type and context-specific, and its generality across other cell types remains to be established. ASIC2a is treated as the buffering subunit, since preconditioning upregulates it [56], and ASIC2a-containing channels have low Ca²⁺ permeability, but ASIC2b/1a heteromers contribute actively to acidosis-induced neuronal death [66]. The buffering effect therefore belongs to a specific splice variant, not the ASIC2 gene product as a class. The hormonal arm is specific in the same way. β-estradiol lowers ASIC1a protein and protects central neurons [91], while 17β-estradiol acting through the same receptor rapidly potentiates ASIC currents in primary sensory neurons [62]. Three variables set the direction of the effect: cell type, timescale, and whether the modulator acts transcriptionally or acutely, and these are the variables the phase-specific framework organizes.

The framework is therefore best read as an organizing hypothesis. Its core premises are well supported. Penumbral acidosis can outlast the initial glutamate surge, and ASIC1a inhibition has retained efficacy when glutamate antagonists are ineffective [58]. Three components remain to be tested. Whether persistent acidosis causes the extended therapeutic window remains unsolved. The pre-stroke arm rests on conditioning protocols that alter channel expression, so testing it requires acute block confined to the protective window. The recovery arm rests on one study of progenitor migration [31] and one behavioural study [3], and would be strengthened by independent replication. The framework earns its place by specifying which experiments would discriminate between phases, a question the field has not previously posed precisely.

## Section IV: future directions and open questions

### Beyond ion conduction: non-conducting role of ASIC1a

ASIC1a can operate in a non ion-conducting way alongside its classical ionotropic function. Sustained acidosis dissociates the cytoplasmic N- and C-terminal domains, exposing a C-terminal death motif that recruits RIPK1 to initiate necroptosis independent of ion conduction [80, 81]. Extracellular protons therefore gate not only channel opening but a downstream signaling cascade. Autoinhibition normally keeps this death motif occluded by the N-terminus. NT1-20, a peptide mimetic that restores the occlusion, was as neuroprotective in MCAO as ASIC1a gene ablation [81]. This non-conducting mechanism offers a possible explanation for injury that develops despite channel desensitization. PKC/PICK1-dependent modulation of ASIC currents has also been reported [6, 65], but whether it influences ASIC1a-RIPK1 conformational signaling remains unclear. Whether these modes operate independently or converge on shared effectors, and whether blocking one suffices or whether both must be targeted, are urgent questions for future work.

### Human versus rodent ASIC: translational considerations

Human ASIC1a (hASIC1a) differs functionally from its mouse ortholog in ways directly relevant to stroke. Human cortex shows a higher membrane-to-total ratio of ASIC1a than mouse, attributable to a single residue substitution at position 285 enabling more efficient N-glycosylation and membrane trafficking [86]. Cells expressing hASIC1a show stronger acid-evoked Ca²⁺ influx and greater acidotoxicity, and human cortex also shows reduced ASIC2b relative to ASIC2a compared with mouse [86]. These differences raise the possibility that rodent models may not fully capture the magnitude or pharmacological profile of ASIC1a-mediated acidotoxicity in human brain. Whether they translate into a quantitative difference in acidotoxic injury during human stroke is not established, since extracellular pH dynamics, buffering capacity, glial expression, and vascular physiology may differ between species and shape the injury response. Further systematic comparisons of ASIC2a, ASIC2b and ASIC3 across species are needed. Since ASIC2a acts mainly as an ASIC1a heteromeric partner, human-specific differences in it could affect the penumbral response in ways current rodent models do not reproduce. A human ASIC1a-selective antibody (ASC06), raised against nanodisc-embedded hASIC1a, reduced infarct volume in MCAO [61], illustrating that human-specific targeting is feasible. More recently, a human ASIC1a-selective nanobody (Nb.h1a) was shown to drive channel endocytosis, lowering surface expression and acid-evoked currents in neurons from humanized ASIC1a knock-in mice [53], which offers a tool for probing human ASIC1a trafficking, though it remains untested in ischemic stroke. Whether drugs optimized against rodent ASIC1a retain efficacy, selectivity and an adequate window against the human channel remains open.

### Non-neuronal cell contributions to ASIC-mediated stroke pathology

The ischemic lesion is multicellular, yet ASIC biology in stroke has focused almost exclusively on neurons. Astrocytes, microglia, oligodendrocyte precursors, and oligodendrocytes all express ASIC subunits [11], but their functional consequences have been characterized almost entirely outside ischemia. In non-stroke models, ASIC1a is low in resting astrocytes but markedly upregulated in reactive astrocytes in rodents and resected human hippocampal tissue [88]. Activation elevates intracellular Ca²⁺ and promotes glutamate release, linking astrocytic ASIC1a to excitotoxic amplification of injury. Whether ASIC1a engagement differs between the neurotoxic A1 reactive phenotype and alternative reactive states [47] is unknown but clinically important. In microglia, ASIC1 and ASIC2a are upregulated by LPS stimulation, and ASIC1a blockade reduces iNOS and COX-2 expression [89]. NG2 glia express high-density Ca²⁺-permeable ASIC1a, and ischemia-related influx may injure the oligodendrocyte precursor pool essential for post-stroke remyelination [48], while ASIC1 contributes to axonal degeneration and demyelination in inflammatory CNS disease [29, 75]. Extending this to stroke, microglial ASIC activation in the peri-infarct zone during the subacute phase may sustain inflammatory mediator production, prolonging secondary injury beyond the acute ionic window, an opportunity not captured by current neuroprotection paradigms. This remains untested in ischemic tissue. Whether ASIC inhibition in non-neuronal cells could extend the window into the subacute phase, when secondary injury may continue alongside tissue repair, is an important question for future investigation.

### Intracellular organelle ASIC expression and function

ASIC subunits are not exclusively plasma membrane proteins. ASIC1a also localizes to the inner mitochondrial membrane of mouse cortical neurons, associating with adenine nucleotide translocase, a core component of the mitochondrial permeability transition pore [79]. *Asic1a⁻/⁻* neurons resist H₂O₂-induced MPT-dependent death, but extracellularly applied PcTx1 did not protect against H₂O₂-induced death in the same experimental system, a gap particularly relevant during reperfusion when oxidative insult is prominent. During occlusion, when the dominant signal is extracellular acidosis, surface ASIC1a is the relevant pool. At reperfusion, oxidative insult is maximal, and mitochondrial ASIC1a may represent a plausible second contributor, acting on a different trigger and through a different effector [79]. The two pools may have distinct functions, and the difference in trigger suggests they may dominate at different phases, though this has not been tested directly. Genetic deletion removes both, while PcTx1, Hi1a, and C5b bind or are predicted to bind the extracellular domain of plasma membrane ASIC1a; whether they can access the corresponding domain and inhibit ASIC1a in mitochondria remains unknown. Any comparison of pharmacological blockade with *Asic1a⁻/⁻* that finds the drug less protective is therefore ambiguous, since incomplete target engagement and an untargeted mitochondrial pool produce the same result. Distinguishing them requires validated compartment-selective approaches.

## Conclusion

Ischemic stroke hijacks the regulatory logic of ASIC channels, converting a transiently active pH sensor into a sustained driver of neuronal death. Acidosis, glutamate, polyamines, intracellular alkalization, and oxidative stress converge in the penumbra to overwhelm the brain’s homeostatic brakes, amplifying injury through both ionic and non-ionic mechanisms. Yet the same family is subject to endogenous constraint, and the balance plays out continuously from pre-stroke through ischemia, reperfusion and recovery.

A temporal phase-specific view therefore provides a useful framework for interpreting these findings and guiding therapeutic development. Within it, the objective shifts from reinforcing endogenous constraint before stroke, interrupting both ionic and non-ionic death signals during acute ischemia, and testing whether ASIC1a inhibition during reperfusion can limit injury associated with residual acidosis. We further propose, as a hypothesis for future testing, that the goal may shift again during recovery toward promoting repair while preserving the plasticity required for rehabilitation. Direct evidence defining the optimal timing, duration and context of ASIC inhibition during recovery is lacking, and this final phase should be regarded as a direction for experimental work rather than an established strategy.

Several questions will determine the trajectory of this framework. It is not established whether the ionotropic and conformational arms of ASIC1a must be inhibited together for complete neuroprotection, nor whether the pharmacological divergence between human and rodent ASIC1a is large enough to compromise clinical translation. The potential of ASIC inhibition in non-neuronal populations during the subacute neuroinflammatory phase has not been systematically interrogated, and the contribution of organellar pools awaits compartment-selective tools. Addressing these questions will expand the conceptual scope and experimental toolkit of ASIC biology, with implications reaching beyond stroke into acidosis-driven disease more broadly. 

## Data Availability

No datasets were generated or analysed during the current study.
